# Importance of Dentistry to the Physician

**Published:** 1890-11

**Authors:** 


### Importance of Dentistry to the Physician.

The extensive sympathetic connection of the dental nerves with the whole system is quite sufficiently manifest to interest the dentist in the study of general physiology and medicine; the physician, too, may vastly add to his knowledge and success in the treatment of many disorders by devoting some special time to the consideration of dentistry proper. While defective teeth in childhood are often evidence of congenital interference with nutrition, later in life they are themselves the frequent causes of many forms of constitutional derangement. Dyspepsia is often unaccountably persistent, and apparently proof against all ordinary treatment, when timely attention to the condition to the mouth might have obviated an obstinate disorder. Neuralgia is too commonly due to a state of the teeth that has been overlooked. Otalgia and otorrhœa are, in many instances, of dental origin. Fistulous openings in the cheeks, that are a life-long disfigurement, might have been averted by heeding the odontological aspect of the case in its early diagnosis. Skin affections and many diseases connected with the nervous centres are not rarely associated with dentition. In fact, nearly every organ of the body may be more or less affected sympathetically by dental lesions; and the delicate organization of the teeth, so much more complex and intricate than that of the compacter structures, renders them singularly liable to diseases that may influence the system and make them worthy of minuter attention from the physician than they ordinarily receive.—L. LEWIS, *The Medical World.*

DR. CURTIS, reports in the *Medical Press,* the case of a young woman suffering from double dislocation of the jaw produced by the act of vomiting.

